# Cap-assisted antegrade pancreatoscopy in surgically altered anatomy

**DOI:** 10.1055/a-2933-0536

**Published:** 2026-08-19

**Authors:** Marcel Razpotnik, Tobias Blasberg, Martin Maibier, Andreas Adler, Silke Leonhardt, Jürgen Hochberger

**Affiliations:** 1 Department of Hepatology and Gastroenterology, Campus Virchow-Klinikum (CVK) 14903 Charite – Universitatsmedizin BerlinBerlinBEGermany

## A case report



**Video 1** Cap-assisted antegrade pancreatoscopy after EUS-guided pancreaticojejunostomy in Billroth II anatomy, demonstrating cap-facilitated advancement across the transmural tract and distal duct stricture, with the assessment of benign stenosis and pancreatic stones. Video URI:


Endoscopic interventions in surgically altered anatomy are challenging and time-consuming. 1​ In this setting, antegrade endoscopic ultrasound (EUS)-guided creation of a pancreaticogastrostomy has previously been associated with high rates of technical and clinical failure. 2​ Its combination with antegrade pancreatoscopy has rarely been described. 3​ Furthermore, advancing the pancreatoscope through a narrow tract or stricture may cause endoscope instability, pushback, and loss of position. 4​

We present a case of cap-assisted antegrade pancreatoscopy in altered anatomy for stricture characterization and pancreatic duct (PD) stone therapy ( Video 1 ).

A 57-year-old man with alcohol-related chronic pancreatitis (Cambridge IV), Billroth II anatomy after duodenal perforation, and an inaccessible papilla presented with worsening upper abdominal pain. Cross-sectional imaging demonstrated a distal PD stricture with suspected stones. After multidisciplinary consultation and refusal of surgery, an EUS-guided approach was anticipated.

**Fig.1:**
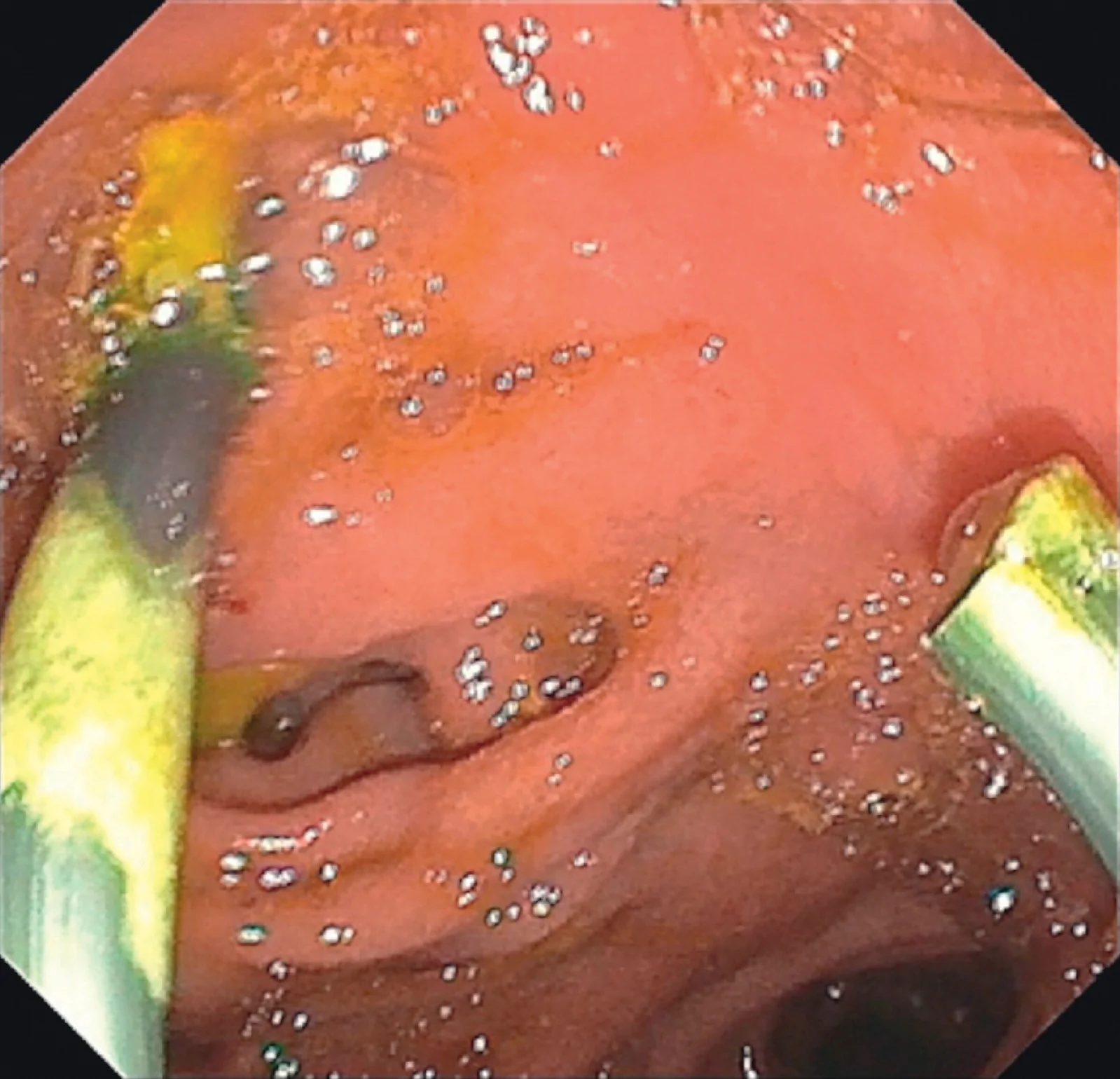
EUS-guided pancreatic duct drainage. A 10 F/12-cm double-pigtail plastic stent was placed into the pancreatic duct approximately 5 cm distal to the gastrojejunal anastomosis.

**Fig. 2:**
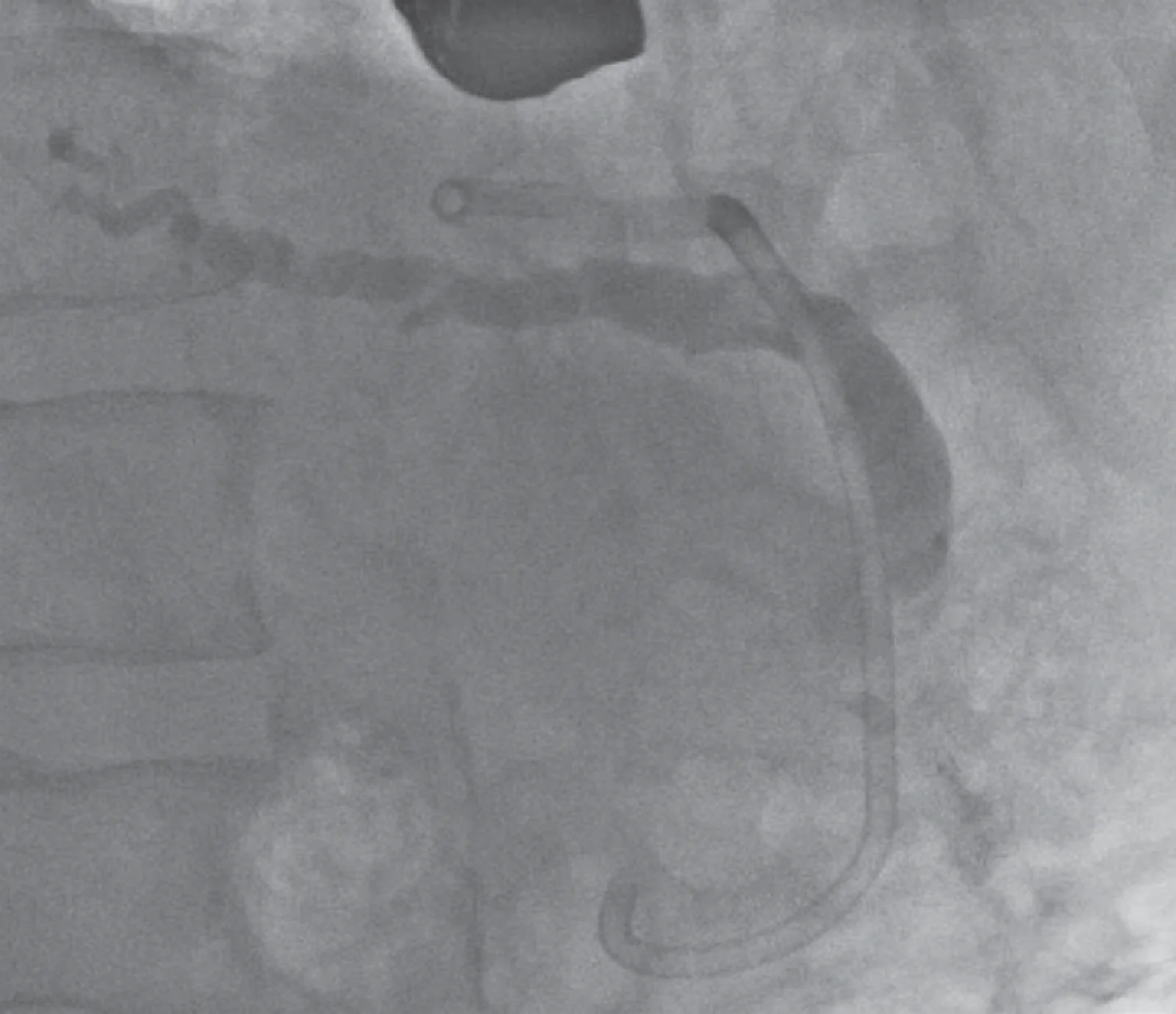
Transpapillary stent position. A fluoroscopic image showing the distal end of the double-pigtail stent crossing the papilla and extending into the jejunum.

**Fig. 3:**
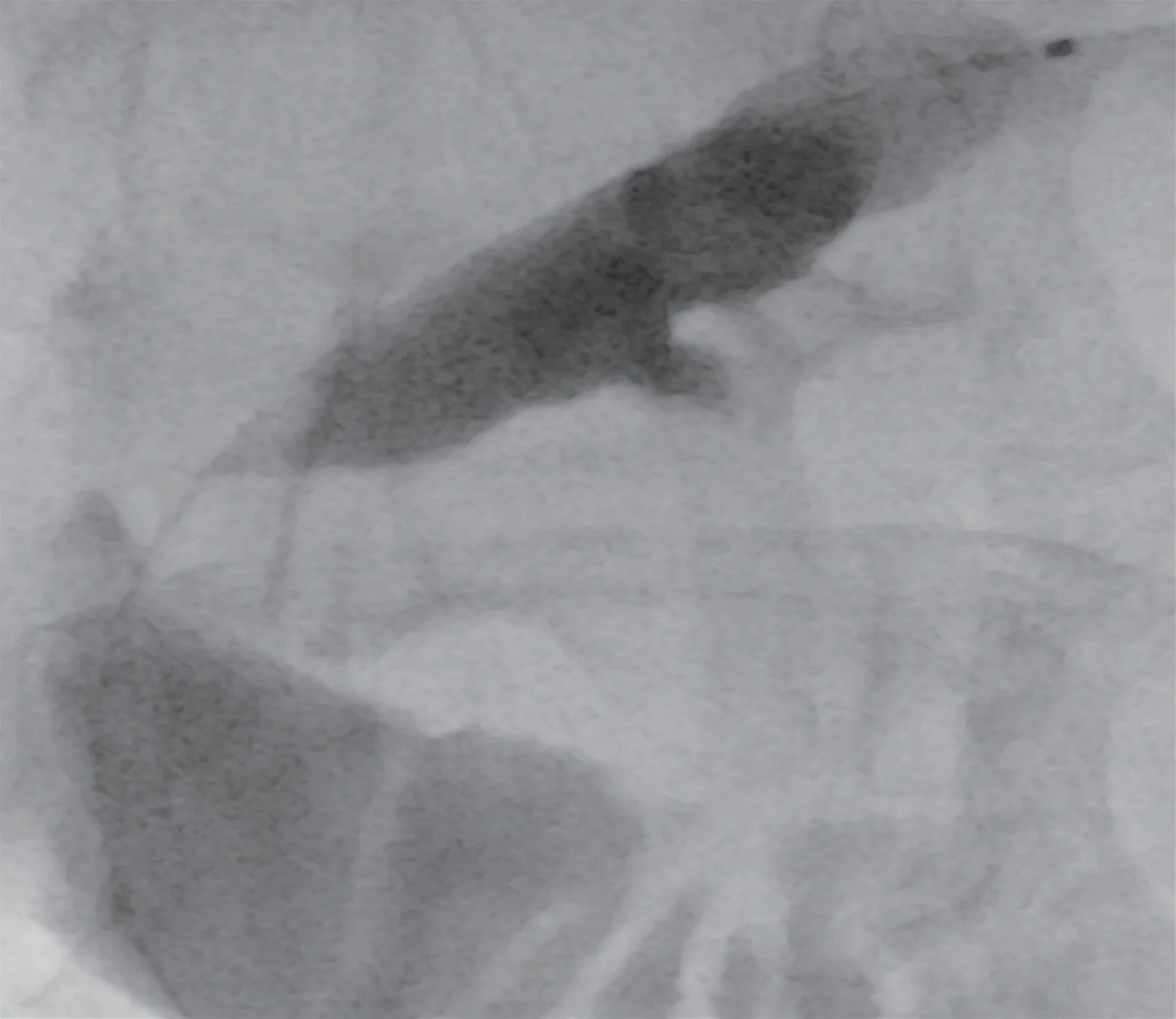
Guidewire passage across the stricture. After stent removal, a catheter-assisted guidewire was advanced under fluoroscopic guidance across the distal pancreatic duct stricture.

**Fig. 4:**
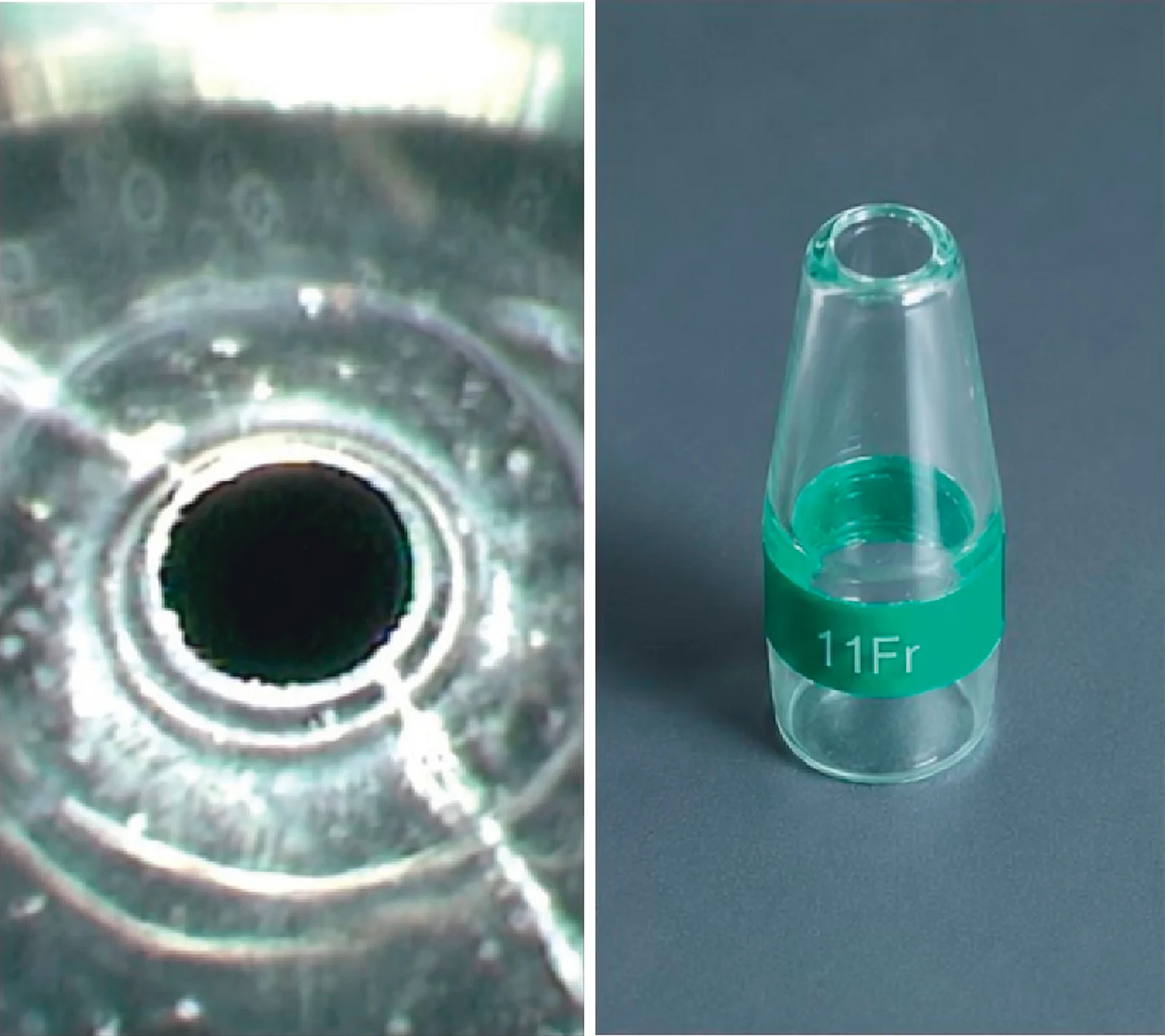
Cap-assisted pancreatoscope insertion. An 11 F conical transparent cap was attached to the pancreatoscope to facilitate passage through the transmural tract and narrowed distal pancreatic duct.

**Fig. 5:**
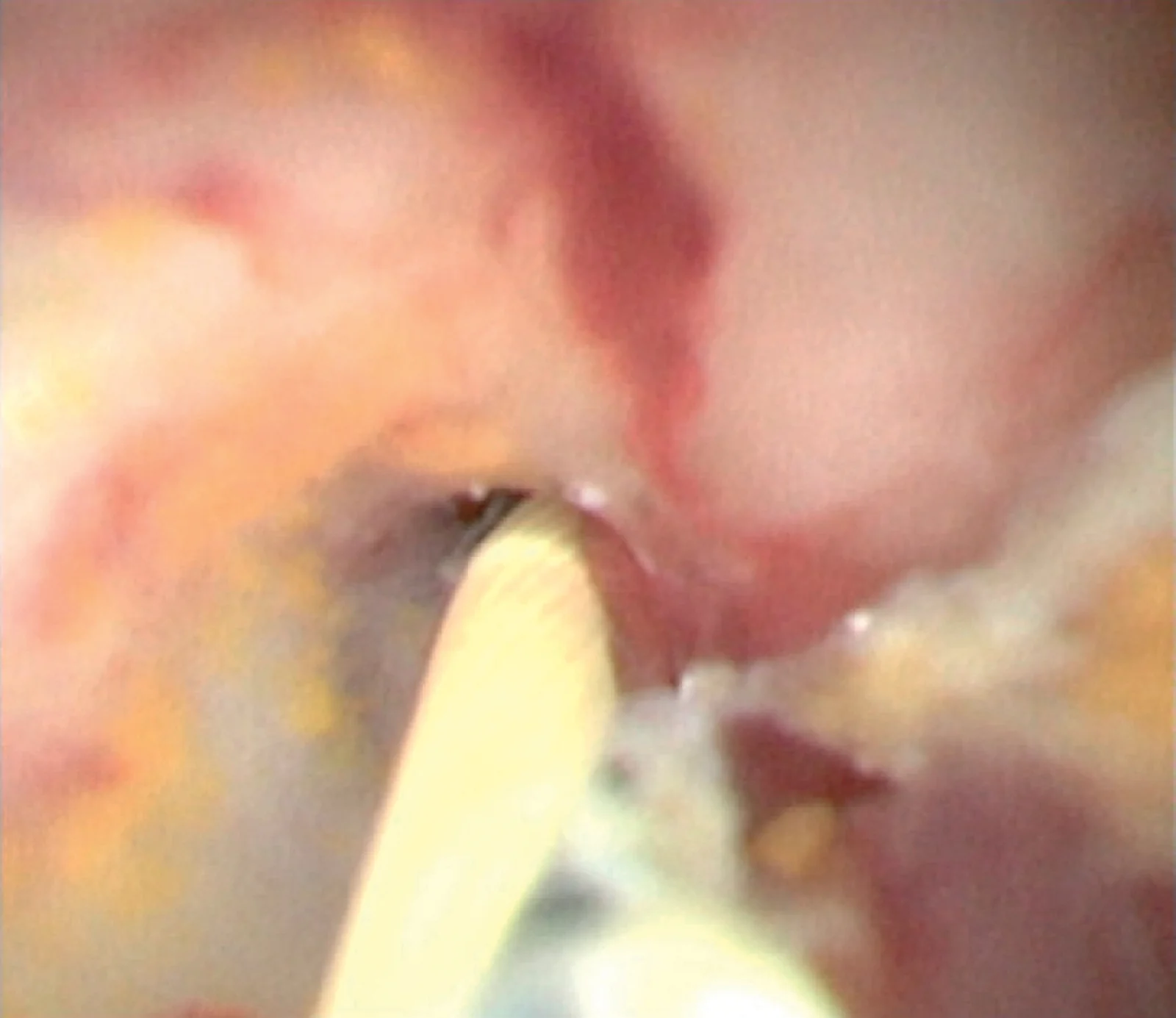
Endoscopic visualization of the pancreatic duct stricture. Cap-assisted antegrade pancreatoscopy enabled the stable close-contact visualization of a benign-appearing distal pancreatic duct stricture.

In the first session, a 10 F/12-cm double-pigtail plastic stent was placed into the PD approximately 5 cm distal to the gastrojejunal anastomosis ( Fig. 1 ), across the papilla into the jejunum ( Fig. 2 ). After 3 months, the patient reported significant pain relief and returned for antegrade pancreatoscopy, stone treatment, and stricture evaluation. The stent was removed with forceps. Under fluoroscopy, a guidewire was advanced through a catheter and passed across the stricture ( Fig. 3 ). To facilitate pancreatoscope insertion through the transmural tract, an 11 F conical transparent cap was attached ( Fig. 4 ). This enabled smooth advancement through the intestinal wall tract and narrowed distal duct without resistance. Close contact provided by the cap allowed good visualization of the benign-appearing stricture ( Fig. 5 ). During withdrawal, only small stones were observed; therefore, lithotripsy was not required. The procedure was feasible and without adverse events.

As the first reported use, this case suggests that cap-assisted antegrade pancreatoscopy may improve transmural access, stability, and visualization. 5​ Further studies are warranted to define its clinical utility.

Endoscopy_UCTN_Code_TTT_1AS_2AI
